# Supplementary material for: KLF10 Inhibits TGF-β-Mediated Activation of Hepatic Stellate Cells via Suppression of ATF3 Expression
Source: Int J Mol Sci. 2023 Aug 9;24(16):12602. doi: 10.3390/ijms241612602 (PMC10454374; doi:10.3390/ijms241612602)
Supplement: Supplementary file 1 [file ijms-24-12602-s001.zip › ijms-2504229-supplementary.pdf]

Supplementary data to International Journal of Molecular Sciences

KLF10 inhibits TGF-β-mediated activation of hepatic stellate cells via suppression of ATF3 expression

Soonjae Hwang [1,†], Sangbin Park [2,†], Uzma Yaseen [2], Ho-Jae Lee [1,2] and Ji-Young Cha [1,2,*]

[1] Department of Biochemistry, Lee Gil Ya Cancer and Diabetes Institute, College of Medicine, Gachon University, Incheon 21999, Republic of Korea; soonjae@gachon.ac.kr (S.H.); hojlee@gachon.ac.kr (H.-J.L.)

[2] Department of Health Sciences and Technology, GAIHST, Gachon University, Incheon 21999, Republic of Korea; tree5267@gachon.ac.kr (S.P.); uzmayaseen255@gmail.com (U.Y.)

* Correspondence: jycha1014@gachon.ac.kr (J.-Y.C); Tel.: +82-32-899-6070

† These authors contributed equally to this work.

Supplementary figure S1. Putative KLF10 binding sites on human and mouse ATF3 promoters.

Supplementary figure S2. KLF10 knockdown does not affect apoptosis in LX-2 HSCs.

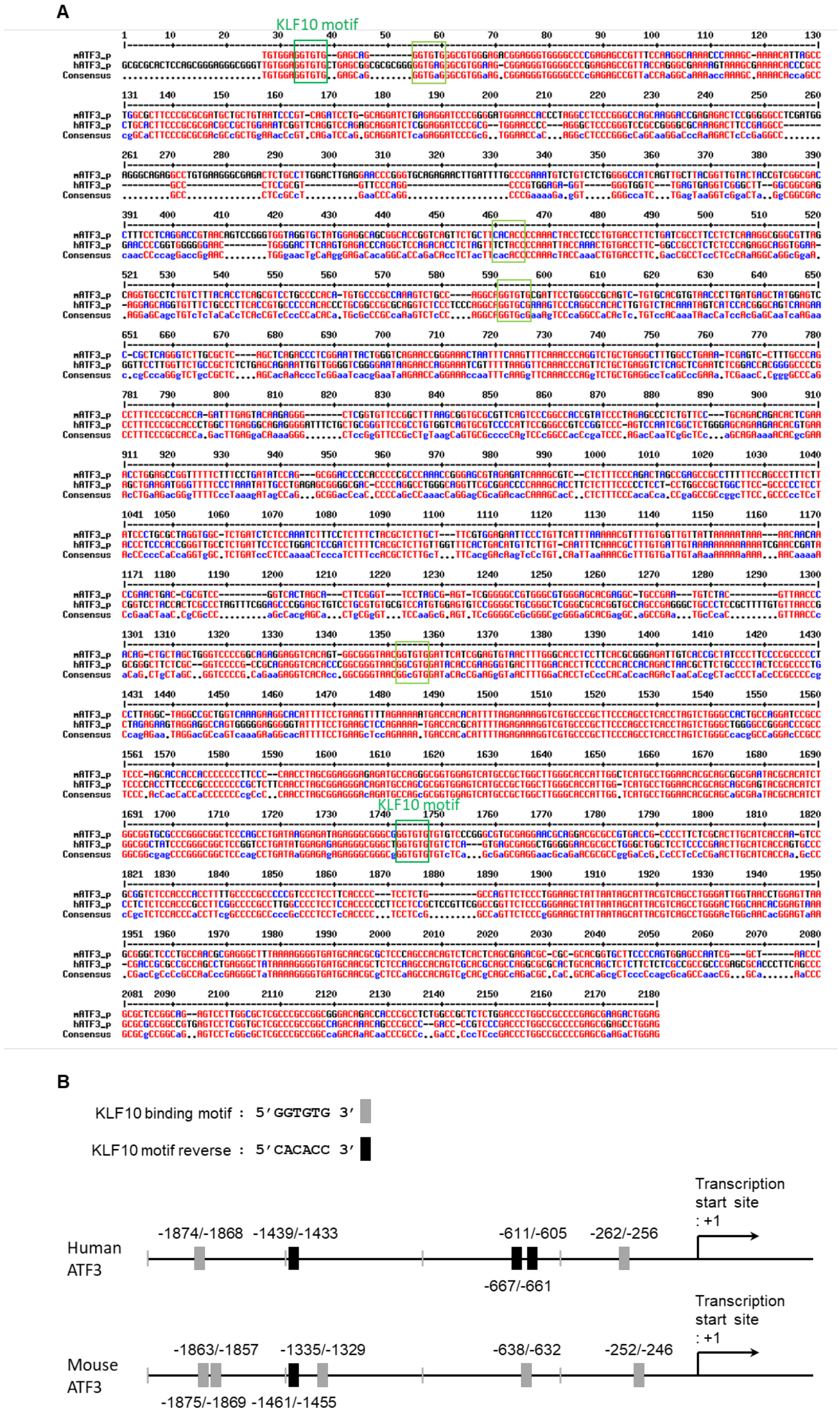
A
KLF10 motif
mATF3_p
hATF3_p
Consensus
KLF10 motif
B
KLF10 binding motif : 5′GGTGTG 3′
KLF10 motif reverse : 5′CACACC 3′
Transcription start site : +1
Human ATF3
-1874/-1868
-1439/-1433
-611/-605
-262/-256
-667/-661
Transcription start site : +1
Mouse ATF3
-1863/-1857
-1335/-1329
-638/-632
-252/-246
-1875/-1869
-1461/-1455

Figure S1. Putative KLF10 binding sites on human and mouse ATF3 promoters. (A) ATF3 promoter alignment in human and mouse. The human and mouse ATF3 promoter regions were retrieved from the EPDnew database (https://epd.expasy.org/epd). Human and mouse ATF3 alignment demonstrated considerable conservation in both species. (B) Localization of the putative KLF10 binding motifs in the human and mouse ATF3 promoters. KLF10 binding motif, 5'-GGGTGT-3' were obtained from the GeneCards (https:// www.genecards.org/cgi-bin/carddisp.pl?gene=KLF10). The promoter alignment and motif localization were examined using Vector NTI 7.1 and JASPAR database.

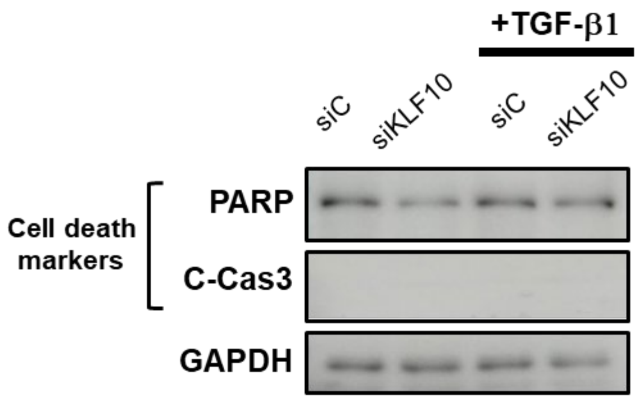

Figure S2. KLF10 knockdown does not affect cell death in LX-2 HSCs. LX-2 cells were transfected with siKLF10 (100 pmol) for 48 h and further treated with 10 ng/mL TGF-β1 for another 24 h. Protein levels of Poly (ADP-ribose) polymerase (PARP) and Cleaved caspase 3 (C-Cas3) were determined by Western blotting.
